# Applying the ROBINS-I tool to natural experiments: an example from public health

**DOI:** 10.1186/s13643-017-0659-4

**Published:** 2018-01-24

**Authors:** Hilary Thomson, Peter Craig, Michele Hilton-Boon, Mhairi Campbell, Srinivasa Vittal Katikireddi

**Affiliations:** MRC|CSO Social & Public Health Sciences Unit, University of Glasgow, 200 Renfield Street, Glasgow, G2 3QB UK

**Keywords:** Non-randomised studies, Controlled before and after, Natural experiments, Public health, Risk of bias, Methodological quality

## Abstract

**Background:**

A new tool to assess Risk of Bias In Non-randomised Studies of Interventions (ROBINS-I) was published in Autumn 2016. ROBINS-I uses the Cochrane-approved risk of bias (RoB) approach and focusses on internal validity. As such, ROBINS-I represents an important development for those conducting systematic reviews which include non-randomised studies (NRS), including public health researchers. We aimed to establish the applicability of ROBINS-I using a group of NRS which have evaluated non-clinical public health natural experiments.

**Methods:**

Five researchers, all experienced in critical appraisal of non-randomised studies, used ROBINS-I to independently assess risk of bias in five studies which had assessed the health impacts of a domestic energy efficiency intervention. ROBINS-I assessments for each study were entered into a database and checked for consensus across the group. Group discussions were used to identify reasons underpinning lack of consensus for specific questions and bias domains.

**Results:**

ROBINS-I helped to systematically articulate sources of bias in NRS. However, the lack of consensus in assessments for all seven bias domains raised questions about ROBINS-I’s reliability and applicability for natural experiment studies. The two RoB domains with least consensus were selection (Domain 2) and performance (Domain 4). Underlying the lack of consensus were difficulties in applying an *intention to treat* or *per protocol* effect of interest to the studies. This was linked to difficulties in determining whether the intervention status was classified retrospectively at follow-up, i.e. post hoc. The overall risk of bias ranged from moderate to critical; this was most closely linked to the assessment of confounders.

**Conclusion:**

The ROBINS-I tool is a conceptually rigorous tool which focusses on risk of bias due to the counterfactual. Difficulties in applying ROBINS-I may be due to poor design and reporting of evaluations of natural experiments. While the quality of reporting may improve in the future, improved guidance on applying ROBINS-I is needed to enable existing evidence from natural experiments to be assessed appropriately and consistently. We hope future refinements to ROBINS-I will address some of the issues raised here to allow wider use of the tool.

## Background

Well-conducted randomised controlled trial (RCT) may be considered to provide the most robust type of evidence for questions of effectiveness. When rigorously implemented, the use of randomisation minimises key sources of bias due to confounding and selection. Consequently, randomisation provides effect estimates which are less susceptible to bias compared to those reported in non-randomised studies (NRS) [1]. However, for many important questions—for example in public health, public policy, and health services research—RCTs are not available or feasible and the best available evidence may come from NRS [2]. Before attempting to use or apply research evidence, it is critical to make an informed assessment about its validity or reliability. All research is susceptible to bias, and any conclusions or lessons should be considered in light of any identified bias or limitations [3]. This is the case whether drawing from a single study or synthesising a body of evidence, for example conducting a systematic review, and also whether or not the evidence is from RCTs or NRSs.

There are many tools available to assess study quality, [4] many of which focus on methodological quality and potentially conflate issues of internal and external validity which is problematic. Over the past few years, there has been a shift to focus on risk of bias. This approach focusses on internal validity, and specific bias domains are considered in turn [5]. In 2011, the Cochrane Risk of Bias (RoB) tool (now updated to RoB v2.0) [6] for RCTs using this domain-based approach was published by the Cochrane Statistical Methods Group and the Cochrane Bias Methods Group. Bias domains included in RoB v2.0 are randomisation, deviation from intended interventions, missing data, measurement of outcomes, and selection of reported result. The Cochrane RoB tool is the preferred assessment method for any RCT included in a Cochrane review, but the tool is not designed to assess RoB for NRS. In October 2016, a new tool to assess RoB in NRS was published: Risk of Bias In Non-randomised Studies of Interventions (ROBINS-I) [5]. This tool was developed by the Cochrane Bias Methods Group, informed by input from a wide international group of leading epidemiologists and methodologists. Publication of this tool represents a potentially substantial improvement in how NRS can be incorporated into well-conducted systematic reviews of interventions; publication of ROBINS-I has been eagerly anticipated by those working in areas where NRS are commonly included in reviews, such as public health. Like RoB v2.0, ROBINS-I focusses on assessing internal validity, assessing seven specific bias domains. Signalling questions (SQs) are provided to help assessors decide the overall assessment for each bias domain. In ROBINS-I, bias is defined as a systematic difference between the results of the NRS and the results expected from a hypothetical target trial which is unrestricted by practical or ethical issues. The rationale for this is that the NRS is attempting to emulate an RCT, and the comparison of the NRS with a hypothetical target trial allows an assessment of the bias in the NRS data relative to a hypothetical RCT addressing the same question. More detail of what the ROBINS-I assessment process involves and the bias domains is provided in Table 1.

**Table 1:** Summary of ROBINS-I tool

**Review protocol level considerations**
Specify aspects of review question: Population, Intervention, Comparator, Outcome (PICO). Outline how complexities in the PICO will be accommodated in the review. Confounders: the review protocol should pre-specify relevant confounders which are related to exposure to the intervention. Co-interventions: the review protocol should state interventions or exposures which are related to the intervention and prognostic for the outcome of interest. **Study level considerations (to be assessed for each study)** Target trial (TT): define key characteristics (eligibility of participants, intervention, comparator, outcome, effect of interest (see below)) of a hypothetical RCT (this should not be limited by pragmatic or ethical concerns). Effect of interest (EoI): per protocol (PP—starting and adhering to intervention as outlined in intervention protocol) OR intention to treat (ITT—assignment to receive intervention regardless of subsequent exposure or adherence) **Bias domains** ***Pre-intervention*** Domain 1: confounding Domain 2: selection of participants into the study At intervention Domain 3: classification of interventions ***Post-intervention*** Domain 4: deviation from intended interventions* Domain 5: missing data* Domain 6: measurement of outcomes* Domain 7: selection of reported result* * Domain also part of RoB version 2.0 for RCTs **Assessment options for each signalling question (SQ)**: Yes, Probably Yes, Probably No, No, No Information. **Domain level RoB assessment options**: Low, Moderate, Serious, Critical, No information. **Overall assessment (by outcome)**: Low, Moderate, Serious, Critical.

As indicated in the ROBINS-I publication, previous versions of the tool have been piloted over its development period. Much of this work has given rise to questions, formally and informally, around the usability of the tool, as well as issues of application to non-clinical topic areas and inter-rater reliability [7–10]. This paper reports the findings of a group of public health researchers experienced in critical appraisal of NRS in applying the ROBINS-I tool to studies of non-clinical public health interventions. Specifically, the studies assessed the health impacts of housing improvement and were previously included in a Cochrane systematic review led by HT [11]. The aim of this work was twofold: (1) to establish ease of use in applying the tool beyond the clinical realm and (2) through informal consensus methods, identify and articulate issues in application of the tool, specifically when applied to studies evaluating the health effects of natural experiments.

## Methods

A group of five public health researchers was convened to use and test the ROBINS-I tool. The level of experience in conducting critical appraisal of NRS ranged from moderate to extensive. All NRS of warmth and energy efficiency improvements [12–16] included in a Cochrane review [11, 17] were selected to be assessed using ROBINS-I. In the original Cochrane review, the studies had been assessed using two tools: The Effective Public Health and Practice Project (EPHPP) [18] tool recommended by Cochrane Public Health (CPH) [19] to assess NRS and the Cochrane RoB tool (version 1.0). In the original review, the Cochrane Effective Practice and Organisation of Care (EPOC) questions on confounding were included in the assessment, and two further items (baseline response and blinding of analysts) were incorporated into the Cochrane RoB tool (version 1.0), to reflect the domains used in the EPHPP tool.

Each member of the group read the ROBINS-I guidance [20] and independently identified queries for wider discussion. The group met to discuss preliminary queries raised, agree to the selected studies to be assessed, and agree to the protocol level considerations.

One study was assessed by each member of the group to identify further areas in need of clarification; developers of ROBINS-I were contacted for clarification on definition of selection bias (Domain 2) before the remaining studies were assessed. All studies were assessed independently by each member of the group. Assessments were entered into a Microsoft Access© database. Assessments for each of the studies were examined by HT for variations by assessor, and three meetings of the assessors were necessary to further discuss and clarify varying interpretations of the questions. Points of common understanding and clarification were recorded and shared with the group to promote consistent assessments across the group. This supplementary guidance document was edited and added throughout the project (Appendix 1).

Finally, we compared the ROBINS-I assessments with the EPHPP and Cochrane RoB (version 1.0) tool assessments from the original review (Appendix 2).

## Results

Of the five studies for assessment, four had a comparison group and one did not. Each of the studies reported outcome measures before exposure to the intervention and at a follow-up period, ranging between 3 months and 3 years, after initial receipt of the intervention. The four studies with a comparison or control group were categorised as having a controlled before and after study design. The following sections describe the protocol level considerations, including the study specific target trials and the RoB assessments for each domain. We report a summary of the discussions within the group and reasons for unresolved consensus around the target trial characteristics and RoB assessments.

### Protocol level considerations

The details of the review question and Population, Intervention, Comparator, and Outcome (PICO) for the review were agreed and are summarised in Table 2. The review PICO was broadly defined reflecting the broad review question (What are the health impacts of warmth and energy efficiency measures?). The outcome selected for assessment was “respiratory health” and included ascertainment by self or parent reported measures. After some discussion, it was agreed that there were no identifiable co-interventions associated with the intervention. Co-interventions are those “that individuals *might* receive”, are “not part of intended intervention”, but are “related to the intervention...and which are prognostic for the outcome of interest” [20]. Disruption experienced during installation of the warmth and energy efficiency measures was considered a possible co-intervention. However, as some disruption is an inevitable part of home improvement, it was agreed that disruption did not meet the required definition of a co-intervention. Relevant key confounding domains considered were baseline health outcome status, housing quality, socio-economic status, and eligibility for intervention. It was agreed that the review question was about the effects of housing improvements as they are delivered in usual practice. Therefore, the EoI was intention to treat (ITT).

**Table 2:** Review protocol considerations and characteristics of target trial (TT) for each study

Protocol/name of included study	Design	Intervention	TT comparison	Effect of interest assessed in study (classification)	Co-interventions/additional co-interventions	Outcome	Participants	Confounders stated in review protocol/additional confounders
			Actual comparison					
Review protocol	–	Warmth and energy efficiency improvements	Eligible for intervention but not in receipt of it	ITT (Classification: baseline)	None	Respiratory health assessed 12 months since intervention (incl self/parent report)	General population eligible to receive intervention	Baseline health; housing quality; socio-economic status; eligibility for intervention
			No intervention/usual care					
Braubach et al. [12]	CBA	Thermal insulation improvements	Eligible for intervention but not in receipt of it	No consensus (Classification: unclear)	Unclear: various additional improvements to communal areas, water, and power supply Not clear if balanced across groups. Mixed views on whether these were “important”.	Asthma attacks in past 3 months	Housing agency tenants	External temperature: contrast between baseline and follow-up despite 12 months later
			No intervention: area not selected for intervention					
Hopton et al. [14]	CBA	Installation of “Heat with Rent” scheme	Eligible for intervention but not in receipt of it	No consensus (Classification: post hoc)	None	Wheeze in past 12 months (parent report)	Children (<16) in social housing	Length of time in house; reason for moving to house
			No intervention: non-participation in “Heat with Rent” scheme					
Walker et al. [16]	CBA	Installation (and possibly repair) of heating system	Eligible for intervention but not in receipt of it	ITT (Classification: baseline)	None	Experience of wheeze in past year (self-report)	Social housing tenants and elderly (> 60 years) private sector households	Age; gender; recent life events; change in smoking exposure; housing type/tenure; central heating at baseline household composition
			No intervention: not eligible for programme (some contamination during study)					
Shortt et al. [13]	CBA	Installation of heating system and other energy efficiency measures	Eligible for intervention but not in receipt of it	No consensus (Classification: post hoc)	Unclear: promotion of welfare uptake to intervention and comparison households. Not reported if balanced across groups. Assessed as “important”.	Asthma (self-report)	Vulnerable groups (>65 years, low income and “infirm”)	Housing tenure
			No intervention: not eligible for programme					
Somerville et al. [15]	UBA	Installation of heating system	NA	No consensus (Classification: NA)	None	Wheeze by day (parent report)	Children (< 16) with asthma in damp social housing	Smokers in household; pets in household; house type; age
			Uncontrolled					

Classification: post hoc is where intervention status is classified at follow-up

Design: *RCT* randomised controlled trial, *CBA* controlled before and after, *UBA* uncontrolled before and after. Effect of interest: *ITT* intention to treat

### Study specific target trial (TT)

Characteristics of the target trials are presented in Table 2. The broad definition of the review question in the protocol meant that, while all studies met the scope and key characteristics of the review, the characteristics of the target trials (TT) were far more specific and highlighted issues of heterogeneity across the study data to be synthesised. Across the five studies, 18 different measures of respiratory health were reported. It was agreed to select a single respiratory outcome for the review protocol to allow agreement with the outcome in the study specific TT; the outcome selected was wheeze. For each TT, the outcome most closely linked to “wheeze” was selected following group discussion. The comparisons in the TTs were those who did not receive the intervention but who were otherwise eligible for the intervention. In the actual studies, the comparison group were those who did not receive the intervention, either through self-selection or failure to meet eligibility criteria, from the named provider specified by the research authors. Although it was possible that individuals received the intervention through other channels as the intervention of interest, warmth improvement is widely available. In the studies, the comparison group, therefore, represented the equivalent of usual care.

### Effect of interest in target trial and post hoc classification of intervention status

The group of assessors was unable to reach consensus about the EoI assessed in four of the included studies. This was largely because in some studies, it was not clear whether the intervention status (i.e. whether a participant was in the intervention or control group) of the study participants was known at baseline or whether intervention status was ascertained at follow-up. This fuelled much discussion about whether or not the concept of ITT or per protocol effects of interest could be applied when the intervention status was classified retrospectively by the research team (i.e. at follow-up, hereafter referred to as post hoc classification) rather than at the baseline period and prior to or at the point of delivery of the intervention (see also the “Domain 3” section below for elaboration about classification of intervention status in ROBINS-I).

### Level of agreement and reasons for lack of agreement in RoB domain assessments

The range of assessed RoB for each study and by domain is presented in Table 3. There was variation across the assessments for each study: this did not appear to be related to particular assessors. Discussions between assessors to clarify common understandings of the signalling questions helped to improve agreement between assessors. However, even assessors with considerable experience in critical appraisal of NRS expressed a lack of confidence in their final assessments. The following section summarises the extent of agreement for each bias domain and where possible identifies explanations for the lack of agreement between reviewers. A summary of key outstanding queries is provided in Table 4.

**Table 3:** Range of overall assessments by study and bias domains

	Domain 1: confounding	Domain 2: selection	Domain 3: classification of intervention	Domain 4: deviation from interventions	Domain 5: missing data	Domain 6: measurement of outcomes	Domain 7: selection of reported result	ROBINS-I overall		Cochrane risk of bias (version1)	EPHPP
Braubach [12]	3–4	(0) 3–4 ^Ŧ^	1	**1**–**4**	2–3	2–3	2	3–4	Serious–Critical	Critical	Low
Hopton [14]	**2–4**	**1–4**	1–2	**1–3**	3	2–3	2	**2–4**	Moderate–Critical	Critical	Moderate
Shortt [13]	3–4	3–4	1–2	2–3	2–3	(0) 2–3^Ŧ^	2–3	3–4	Serious–Critical	Critical	Moderate
Somerville [15]	4	3–4	1	1–2	2–3	3	2	4	Critical	Critical	Moderate
Walker [16]	2–3	**1–4**	1–2	1–2	(0) 2–3^Ŧ^	**1–3**	2–3	2–3	Moderate–Serious	Critical	Low

Risk of bias assessment: 0 No information; 1 Low; 2 Moderate; 3 Serious; 4 Critical

Bold figures indicate disagreement of two or more levels of bias across assessments

^Ŧ^0 (no information) was assessed as equivalent to “Serious” (3) indicating agreement within one level of bias for each domain where “0” was used by one or more assessor

**Table 4:** Summary of outstanding queries for Domain 2 and 4 of ROBINS-I tool

**Domain 2: selection of participants into the study**
• Further guidance on the distinction between SQ2.2 and SQ2.3, (“Were the post-intervention variables that influenced selection: likely to be associated with the intervention (SQ2.2); OR likely to be influenced by the outcome or a cause of the outcome (SQ2.3)”. For analysis relying on post hoc classification of intervention status, this may be difficult to assess but may be a critical source of bias. Also, for interventions that address socio-economic determinants of health, it is highly likely that selection to receive the intervention will be on variables such as income or other measures of socio-economic deprivation, which is also a determinant of the outcome, health. • Start of intervention coinciding with start of follow-up: clarification on how this should be assessed for studies where a baseline assessment of the outcome was made before the participants received the intervention and then at follow-up after the intervention. • Can SQ2.4 (Do start of follow-up and start of intervention coincide for most participants?) be applied to non-event type outcomes? • Clarification of whether variation in lengths of exposure to the intervention at follow-up could contribute to selection bias. **Domain 4: deviation from intended interventions** *Effect of interest* • Can analysis of post hoc classification of intervention status be interpreted as per protocol? • Clarification of question “If your aim for this study is to assess the effect of initiating and adhering to the intervention (as in a per protocol analysis)”. Does this relate to the aim for the review as agreed in the review protocol characteristics or the aim of the analysis used in the study being assessed? *Implementation and adherence* • Clarification about what is meant by successful implementation (SQ 4.4) and how authors should decide the level at which implementation failure (SQ 4.4) and adherence (SQ4.5) is assessed (see Table 5). • How should interventions which are tailored to individual need be assessed? • Can it be assumed that there is no implementation failure where classification of intervention status is post hoc? By definition, all those in the intervention group may be assessed to have received the intervention, but this will be dependent on the level at which the intervention is being assessed (see Table 5). *Co-interventions* • Clarification about what constitutes a co-intervention (see Table 5). • Clarification about when a co-intervention should be considered to be “important” (SQ4.3). Should there be an established association with the outcome? *Contamination and switching* • Clarification about the meaning when “contamination” constitutes “switching”, especially in cases where classification of intervention status is post hoc. Guidance implies that contamination is inadvertent but this is difficult to determine in studies, see page 35 of ROBINS-I guidance.

### Domain 1: confounding

Confounding variables considered to be relevant at the protocol level were assessed as critical. If the critical confounders were not taken into account through study design or analysis, this resulted in a “critical risk of bias” assessment due to confounding. Additional confounders for individual studies were considered to be critical where there was clear justification in the paper.

Most assessments for Domain 1 were within one degree of each other. The variation in assessments was largely explained by differences in assessors’ interpretations of the potential for bias from identified confounders and adequacy of adjustment for confounders.

### Domain 2: selection of participants into the study

Domain 2, together with Domain 4, had a high level of variance between assessors which was not fully resolved through discussion. Confusion about what “selection into the study” (Q2.1) meant led to a query to the developers of the ROBINS-I tool. Following this, guidance was developed to promote consistent assessments, and an alternative signalling question (SQ) for SQ2.1 was provided (Appendix 1). The distinction between prevalent and incident exposure was emphasised to assessors, and it was highlighted that differences in eligibility and inclusion in the analysis was the issue of interest rather than selective inclusion for the intervention, study, or dataset. However, discussion about selection for the intervention, the study, and the analysis were not completely resolved. In addition, it was not always clear from the study report whether or not selection for the intervention and classification of intervention status, the study, and/or the analysis was post hoc (Table 2, see Braubach et al. [12]). The variations in assessment also drew attention to differences in assessors’ interpretation of the level at which the intervention was being assessed: delivery of the programme at an area level, delivery of the intervention to households, or implementation and adherence to the intervention by householders (see Table 5).

**Table 5:** Differing interpretation about level of intervention being assessed and related implementation failure

**Provided below are quotes from two included studies on study aim(s) and interventions**
**Braubach** [12]* Study aim: “to assess potential health impacts of improved thermal insulation. Key objectives were to assess the impact of thermal insulation changes on indoor environments, and evaluate potential effects on residents’ health.” ***Intervention description (may include possible co-interventions)***: “thermal insulation of all building facades; thermal insulation of the roof/ceiling of highest dwelling; thermal insulation of basement/floor of lowest dwelling; installation of energy-efficient windows where replacements were necessary; installation of new heating systems in buildings with substandard systems. Additional renovation projects without significance for thermal comfort were painting of staircases, installation of intercom systems, new power and water supply systems, improvement of outside spaces/greenery and other repairs as required. However, these renovations are not part of the survey and their impact will not be looked at, although they may improve the general quality of the dwelling significantly.” **Shortt** [13]* Study aim: “The evaluation focussed on two elements of the process firstly, assessing the benefits to households in terms of indoor environment, health and wellbeing and household income…. This paper focuses primarily on the installation of central heating in selected households and the immediate effect on the dwellings and their occupants” ***Intervention description (may include possible co-interventions)***: “installation of central heating systems and other energy efficiency measures in homes…The overall aim was to develop an energy efficiency programme in partnership with key agencies and local communities and as a result to increase energy awareness, increase uptake in grants and reduce the adverse effects on health and well-being caused by cold homes.” The intervention also involved “encouraging higher uptake of social security benefits.” *In both the above examples, the intervention was tailored according to the need of the individual household, but details of this was not reported and was not controlled for in the analysis. In addition, subject to participants’ own resources, the interventions were available to participants regardless of participation in the study, raising the potential for contamination. Again, this was not reported on. Possible levels of implementation: potential for implementation failure/variation in adherence** ***Programme level***: selective uptake by eligible households or by external factors e.g. changes in funds available to those delivering intervention ***Operational level***: incomplete delivery or installation of intended intervention(s). Successful implementation may also require an educational component to ensure recipients know how to use intervention effectively and have aspects such as potential benefits and costs explained. ***Household level***: householders in receipt of intervention do not use intervention as intended—heat more rooms but with same cost. Impacts are dependent on behavioural change. **External factors can also affect implementation and/or adherence such as changes in fuel costs.

Assessors queried the distinction between SQ2.2 and SQ2.3, which asks whether the post-intervention variables that influenced selection were likely to be associated with receipt of the intervention (SQ2.2) or likely to be influenced by the outcome or a cause of the outcome (SQ2.3). For interventions that address socio-economic determinants of health, and where the evaluation allows for post hoc classification of intervention or control status, it is highly likely that selection into the intervention group may be influenced or even determined by health status (e.g. investing in warmth improvements amongst people who have a household member with asthma) or causes of health status (such as income or other measures of socio-economic deprivation). There is a further risk of selection bias as the studies or evaluations were conducted on discrete populations likely to be offered the intervention. It is therefore possible that participation in the survey or study could be perceived by potential participants to be associated with receipt of the intervention, and/or health status may have influenced participation; hence, the sample analysed within the study may provide misleading estimates of the EoI.

Each of the studies were conducted prospectively and assessed the effect of incident exposure. Baseline assessment of the outcome was made before the participants received the intervention and then at follow-up between 3 months to 3.5 years after the intervention. Assessors were not in agreement about whether this meant that the start of the intervention and start of follow-up coincided (SQ 2.4) and whether SQ2.4 could be applied when time-to-event (survival) analysis was not being used. The ROBINS-I guidance refers to time-to-event outcomes that are typically assessed using survival analysis (e.g. death, incident disease, etc.), but in our studies, the outcomes were assessed using repeated measures (e.g. asthma symptoms) on a panel or cohort of participants.

In most studies, the lengths of exposure to the intervention at follow-up varied within the study sample. In one study, the variation was over 2 years. Some assessors raised this as introducing selection bias as well as being related to assessments about the start of intervention coinciding with follow-up. However, following discussion, it was agreed not to treat this as a component of selection bias. There was further discussion about whether the least possible RoB for this domain for studies with no control group would be “Serious”.

### Domain 3: classification of interventions

Domain 3 had the greatest level of agreement, with all assessments within one degree of each other. Based on the ROBINS-I guidance, our assessments focussed more on differential misclassification of interventions than the timing of recording intervention status. The guidance on misclassification of interventions emphasises potential bias due to recall bias or retrospective identification of eligible participants. The issue of post hoc classification of intervention status is likely to be an important consideration of this domain; this could be clearer in the guidance and signalling questions.

### Domain 4: deviations from intended interventions

There was a high level of variance in assessments across assessors for Domain 4. The different assessments arose mainly for the following reasons: confusion about the EoI; how to assess studies where the intervention status was classified at follow-up; post hoc classification; and a lack of clarity about the meaning and appropriate application of key concepts for this domain, such as implementation or adherence, co-interventions, contamination and blinding.

There was confusion about whether the question leading into SQs 4.2–4.4 (if your aim for this study is to assess the effect of initiating and adhering to the intervention (as in a per protocol analysis)) related to the aim for the review and the characteristics of the review outlined at the “protocol considerations” stage (see above) or the type of analysis which had been used in the study being assessed. This also raised questions about whether analyses based on post hoc intervention classification could be described as ITT or per protocol.

There was uncertainty among assessors about what was meant by successful implementation (SQ 4.1), and the level at which implementation failure (SQ 4.1) and adherence (SQ4.2) should be assessed. Should this be implementation of the programme, or delivery of the intervention locally or use of or adherence to the intervention by household? (Table 5) Or should it involve an assessment at all levels? And how should interventions which are tailored to individual need be assessed? Regardless of what level is to be assessed, implementation difficulties were almost impossible to assess due to lack of reporting. It was also suggested that, where intervention classification is post hoc, it may be assumed that there is no implementation failure, as by definition, all those in the intervention group have been assessed to have received the intervention.

There was uncertainty about what might constitute a co-intervention and about when a co-intervention should be considered to be “important” (SQ4.3). Reference to the definition of a co-intervention in the supplementary guidance (Appendix 1) enabled increased agreement between assessors. Some uncertainty persisted, in particular, where studies focussed on the impacts of housing improvement delivered to individual households as part of a broader programme of neighbourhood improvements. In such cases, the additional intervention(s) were part of the intended intervention, so may not be a co-intervention, but the additional intervention may be related to the outcome. For example, in one study, it was reported that there were various additional renovations to communal areas and changes in water and power supply delivered to some households. Although these changes may be related to the health outcome of interest, the authors of that study reported that these additional changes were not relevant for changes in thermal comfort [12] (Table 5). Our group of assessors was unclear whether this was a co-intervention and whether it should be considered as “important. In another study [13], the programme being delivered included promotion of welfare uptake. Uptake of this part of the intervention was greater in the comparison group, and household income increased more in the comparison group than in the intervention group. In most cases, it was not always known what proportion, far less which individuals, had received the additional intervention(s), and the additional intervention(s) were sometimes available to the comparison group.

There was also lengthy discussion about when “contamination” constituted “switching” and when it should be considered as time varying confounding under Domain 1. The confusion arose partly due to different interpretations of the ROBINS-I guidance, as well as a lack of clarity about the level at which the intervention was being assessed (Table 5). In one study of a heating intervention which used an ITT analysis, 7.2% of the intervention group did not receive the intervention of interest during the study period, despite being exposed to the programme of housing improvements, while 25.7% of the comparison group had heating measures installed during the study period [16]. This was assessed to be “contamination” by some but not all. Further, there was unresolved discussion about the interpretation of “switching” where intervention and comparison groups were classified post hoc rather than at baseline. Inadvertent changes in exposure to the intended intervention or usual care may still occur where the intervention classification is post hoc: However, this is not always assessed or known, especially where the intervention, such as domestic heating measures, is widely available to participants through other sources, rather than being restricted as many clinical procedures and prescriptions are.

### Domain 5: missing data

There was a high level of agreement for this domain with all assessments being within one degree of each other. A threshold for completeness of data is not provided by the ROBINS-I guidance. It was suggested that an 80% threshold may be useful for our studies given that the outcome of interest was not rare, and attrition was unlikely to be related to our intervention of interest. There was discussion about the extent of RoB due to attrition being related to intervention effect size. If the effect on the outcome is large, the effect of an identified RoB due to attrition may be less important than on a small reported effect on the outcome. For our intervention and outcome of interest, none of the effects were expected to be large. This discussion was not pursued to the point of defining “large” and “small” effects as one of the ROBINS-I developers advised us that small effects were not more susceptible to bias and that the balance of missing data across groups was more important for Domain 5. It was unclear how to assess the balance across groups for an uncontrolled study.

### Domain 6: measurement of outcomes

Assessments for all but one study were within one degree of variation for Domain 6. The assessment for this domain focusses on the use of objective outcomes and blinding of assessors (SQ6.1 and 6.2). It was difficult to assess the overall level of bias introduced in this domain, given that self-reported outcomes were included as an outcome of interest in the review protocol and the assessors agreed that blinding to the intervention would not occur. In addition, there were varying assessments of the subjectivity of different self-reported measures. For example, self-report of a diagnosis of asthma may be considered to be less subjective than self-reported wheeze.

### Domain 7: selection of reported result

There was a high level of agreement for this domain with all assessments being within one degree of each other. It was agreed that where there was no protocol for the study, the least severe assessment possible for SQs7.1 and 7.2 would be “Probably Yes”.

### Overall assessment

The overall assessment for our studies varied, with most assessments being “Critical” or “Serious” (Table 3). The overall assessment for any single study cannot be less severe than the most severe assessment allocated for a single domain for that study. Domain 1 (confounding) was the highest (greatest RoB) scoring domain, meaning that the overall assessment largely reflected the level of confounding assessed. There was greater agreement for the overall assessment than for the individual domains; there was only one degree of difference in the overall assessment for 4/5 studies.

### Assessing direction of bias

A question about the direction of bias is an option at the end of each domain and the overall assessment. However, the group of assessors agreed that it was not possible to assess this as no clear rationale to support these judgements was identified.

### Comparison of ROBINS-I with EPHPP and Cochrane RoB tools

While there is some overlap across the tools, there are also important differences in the bias domains assessed (see Appendix 2). This limits the scope for a detailed comparison across the three tools, and for this reason, only the overall assessment for each study was compared (Table 3). From this small group of studies, it would appear that ROBINS-I detected a higher RoB and may also allow for greater nuance in the detection of bias compared with the EPHPP tool. The Cochrane RoB assessments were all “critical”, perhaps reflecting the absence of randomisation, while there was variation in the ROBINS-I assessments of bias across the studies.

## Discussion

This work applied the ROBINS-I tool to a collection of housing improvement studies. We aimed to assess applicability and articulate the issues encountered when applying ROBINS-I to a complex non-clinical intervention delivered in a community or “usual care” setting, particularly when applied to studies using a controlled before and after (CBA) design. ROBINS-I helped to systematically articulate sources of bias in NRS; however, the lack of consensus in assessments raises questions about its reliability. In particular, there may be useful generalisations when interventions are assessed in a“usual care” setting using a CBA design, and where the EoI does not readily conform to ITT or per protocol.

Some of the difficulties with applying ROBINS-I to CBAs of natural experiments may be resolved through greater clarity in the guidance (see Table 4 for suggestions based on our experiences) and provision of examples from non-clinical interventions would help non-epidemiologist researchers to grasp important concepts underpinning the tool and the RoB domains. However, we identified more fundamental difficulties related to the underpinning concepts of ROBINS-I, which are discussed in more detail below.

### ROBINS-I for natural experiments: effect of interest (EoI), post hoc classification of intervention status, and appropriation of target trial

ROBINS-I assumes that the EoI being assessed in studies under review will clearly be ITT or *per protocol*. When, as in our experience, this is not straightforward, use of the ROBINS-I tool is highly problematic. The EoI for the original review [11] was ITT. The review question was about the effects of an intervention being delivered in a “usual” care setting rather than efficacy. Therefore, our EoI was of initiating an intervention or being allocated to an intervention (ITT), rather than the effects of adhering to an intervention (*per protocol*). Our group of studies appeared to assess the effects of initiating an intervention in “usual care” and the unit of analysis was an individual within the household. However, ambiguity about the unit or level of allocation of the intervention (Table 5) and the appropriate level of assessment for implementation of and adherence to the intervention, as well as lack of clarity about the time of classification of intervention status, presented difficulties when attempting to decide and agree an EoI, either ITT or *per protocol*. The use of post hoc classification of intervention status further complicated attempts to agree the EoI as well as raising issues of selection and performance bias. Our studies gathered data prospectively, with no apparent selection of a sub-group for the main analysis. Despite this, it is possible that individuals self-selected into or out of the intervention group during the study period for reasons that are linked to the intervention and the outcome. This will be impossible to determine when intervention status is classified post hoc and therefore introduces the possibility, albeit unknown, of critical selection bias (Domain 2), as well as switching and time-varying confounding (Domain 4).

The studies assessed fitted the definition of “pragmatic trials”, assessing effects of an intervention in the “usual care” setting [21]. Despite this, it could be argued that these studies were not trials, neither pragmatic nor explanatory. Consequently, these studies were not setting out to establish effectiveness and the size of an effect; rather, the purpose of these studies was to make use of naturally occurring interventions, or natural experiments, [22] to identify the existence, nature, and direction of hypothesised health effects. These studies of natural experiments are, therefore, at an earlier, more exploratory stage, with different evidence priorities than is implicit in the justification for a clinical trial, where evidence on basic issues of safety and impacts on key outcomes is already available. The exploratory and opportunistic nature of these studies, together with a possible limited amount of epidemiological or trial input to the study, may also explain why key issues such as EoI, level and a clear definition of the intervention being assessed, timing of classification of intervention status, and unit of assessment for analysis are not always clearly articulated by study authors. This raises important questions about the nature of questions being addressed by these studies, as well as the nature of questions that evidence syntheses of data from these more exploratory studies can address. This may also have implications for the application of RoB assessments.

### Applicability and usability of ROBINS-I for controlled before and after studies of natural experiments

The difficulties in applying some of the concepts which underpin the ROBINS-I tool to this group of studies, as well as the poor levels of inter-rater reliability, raise questions about the applicability of ROBINS-I to assess RoB in evaluations of natural experiments. To counter this, one of the studies we assessed did perform an ITT analysis, [16] suggesting that issues of post hoc classification and a clear EoI should not be regarded as a defining characteristic of CBAs of natural experiments. Moreover, issues of reliability are common in critical appraisal tools and are certainly not unique to ROBINS-I [23–28]. Improvements in reporting of intervention details [29, 30] as well as timing of classification of intervention status and EoI in primary studies could facilitate improved applicability of ROBINS-I to CBA studies in public health, but this will take years to be widely implemented. In the meantime, there remains a need for a usable tool to assess RoB of published evidence in CBAs of natural experiments and which can take account of the issues outlined above.

ROBINS-I has been carefully developed and incorporates complex epidemiological concepts. Use of the tool may require a level of epidemiological knowledge which is beyond the capacity of many systematic review author groups. Based on our own experience, where all assessors had at least some experience in critical appraisal of NRS, ROBINS-I was difficult to apply and not always helpful in providing a sensitive assessment of RoB in CBA studies of natural experiments. In addition, the investment needed to apply ROBINS-I may be of questionable value when it is known beforehand that there are important sources of bias in the studies and when the data and subsequent synthesis are not expected to produce conclusions with high levels of certainty around a precise effect estimate. In ROBINS-I, the overall RoB for a study is determined by the highest level of bias in any single RoB domain. In our small group of studies, the overall RoB was determined by Domain 1 (confounding). One suggestion to improve efficiency in applying ROBINS-I would be to conclude the assessment when any domain is assessed as “Critical”. However, others have raised questions about the appropriateness of applying stringent RoB standards to natural experiments. Specifically, suggesting that a RoB tool which results in all evidence being labelled as “Critical” RoB may hinder the development of evidence and knowledge for many important topic areas, in particular within public health [31]. This, together with earlier questions about comparing these studies to trials and determining ITT or per protocol EoI, points to the need to explore the distinct contribution of data from natural experiments and how they may be used in subsequent evidence syntheses.

## Conclusion

The ROBINS-I tool is a conceptually rigorous tool which focusses on risk of bias due to the counterfactual and consequently articulates limitations in the assessed studies with respect to causal effect. Acknowledging these possible sources of bias is critical and an issue which has not been well addressed in previous critical appraisal tools. However, currently, ROBINS-I is difficult to apply: ROBINS-I and its guidance require further modification if it is to be applied appropriately and reliably to studies assessing the effects of natural experiments. The ROBINS-I developers are currently working to improve the applicability of the tool to specific types of NRS, e.g. controlled before and after, interrupted time series, and regression discontinuity designs. We hope that the issues outlined in this paper, in particular clarification about the appropriate EoI where the timing of intervention classification is unclear or post hoc, will be addressed in future versions of ROBINS-I.

## Appendix 1

### Supplementary guidance developed by authors to facilitate consistent assessments

#### Domain 1: confounding

Confounders should be fitted into broad domains where possible (see protocol level domains). Protocol level confounders should be considered as critical. Additional confounders should only be added where there is a grounded justification to include this as an additional confounder, i.e. not simple speculation that something may have happened.

#### Domain 2: selection of participants into the study

SQ2.1 “Was selection into the study (or into the analysis) based on participant characteristics observed after the start of the intervention?”: This does not mean selection of area or household or individual for intervention or selection into study. Rather, it means selection for analysis at end of study. Alternative question “Was the sample in the final analysis different to original study sample with respect to outcome or exposure to the intervention (aside from attrition)?”

SQ2.4“Do start of follow-up and start of intervention coincide for most participants?”: Criteria to define what is meant by start of intervention and follow-up coinciding is not necessary. This is about loss of important data before first follow-up.

Variation in length of exposure to intervention is not part of selection bias.

None of the studies looked at prevalent exposure, rather, they assess incident exposure. So all studies will be Y/PY for SQs 2.1 and 2.2 (NB: this was not adhered to by assessors).

Note the overall assessment implies that 100% response is required to be assessed as “low”; it is therefore unlikely/impossible for any study to be assessed as “low” for this domain. (NB: this was not adhered to by assessors).

#### Domain 3: classification of interventions

SQ3.2 “Was information used to define intervention groups recorded at the start of the intervention?”: This relates to misclassification of intervention status in analysis and less about timing of recording intervention status.

SQ3.3 “Could classification of intervention status have been affected by knowledge of the outcome or risk of the outcome?”: This relates to those in the study sample.

#### Domain 4: deviation from intended interventions

Disruption is not a co-intervention; rather, disruption is an essential part of the intervention. In ROBINS-I guidance a co-intervention is “a new intervention that is not part of intended intervention”, and “that individuals *might* receive”, and “related to the intervention...and which are prognostic for the outcome of interest”. Bear in mind that questions assume potential for co-interventions to be balanced across groups, and if not, this introduces bias.

For study specific target trial, all additional interventions which were part of the intervention but where it was not an essential part of the intervention should be listed, e.g. where welfare advice or energy efficiency advice was also offered but not always taken up.

SQ4.5 Switching includes contamination, though there appears to be inconsistent advice in the guidance, see p22 and p35 of guidance.

#### Domain 5: missing data

SQ5.1 “Were outcome data available for all, or nearly all, participants?”: No threshold for completeness is provided. It may be useful to have a threshold to improve transparency and consistency of assessments. As none of the outcomes of interest are rare and it is unlikely that attrition will be related to our intervention of interest, an 80% threshold may be appropriate.

Balance of missing data across intervention and comparison group is more important than taking effect size into account.

#### Domain 6: measurement of outcomes

No additional guidance

#### Domain 7: selection of reported result

SQ7.1 “Is the reported effect estimate unlikely to be selected, on the basis of the results, from multiple outcome measurements within the outcome domain?”and Q7.2 “Is the reported effect estimate unlikely to be selected, on the basis of the results, from multiple analyses of the intervention-outcome relationship?” If no protocol maximum assessment for both questions will be “Probably Yes” (NB: this was not adhered to by assessors).

## Appendix 2

**Table 6:** Comparison of Cochrane risk of bias (RoB) version 1.0, Effective Public Health Practice (EPHPP) tool, and ROBINS-I bias domains assessed

Type of bias assessed	Cochrane risk of bias (RoB) domains	EPHPP tool domains	ROBINS-I domains	Comment
Selection	Sequence generation (Cochrane RoB)			Not applicable to NRS
	Allocation concealment (Cochrane RoB)	Study design (EPHPP)	Domain 1: confounding and Domain 2: selection	
Confounding	Baseline outcome characteristics similar (EPOC)	Control for confounding through analysis or design (EPHPP)	Domain 1: confounding	
	Baseline characteristics similar (EPOC)			
Baseline response	Baseline response (EPHPP)	Selection (EPHPP)		
Attrition	Incomplete outcome data (Cochrane RoB)	Withdrawals at follow-up (EPHPP)	Domain 5: missing data	
Contamination*	Contamination (EPOC)			
Reporting*	Selective reporting (Cochrane RoB)		Domain 7: selection of reported result	
Performance *	Blinding—participants (Cochrane RoB)	Blinding—participants and assessors (combined) (EPHPP)	Domain 6: measurement of outcome	Rarely applicable to housing improvement studies—no studies blinded participants
Detection*	Blinding—assessors (Cochrane RoB)			
	Blinding—analysts (EPHPP)			
Performance*	Intervention implementation: within study variation of exposure to intervention (review authors)	Heterogeneity of exposure to intervention and potential to benefit from intervention (Review authors)	Domain 3: measurement of intervention	This measure used in the Cochrane RoB and EHPP was developed by the authors
			Domain 4: deviation from intended intervention	
Outcome measure*		Data collection (EPHPP)		Designed to indicate appropriate data collection tools and outcomes

Bracketed text indicates source of item: *Cochrane RoB* mandatory Cochrane RoB items; *EPOC* additional Cochrane risk of bias items recommended by the Effective Practice and Organisation of Care (EPOC) group; *EPHPP* EPHPP tool recommended by Cochrane Public Health group

*Bias items NOT used in assessment of overall study quality for the original review of housing improvements due to lack of variation or application, e.g. no studies were blinded

## Abbreviations

CBA: Controlled before and after
CPH: Cochrane Public Health
EoI: Effect of interest
EPHPP: Effective Public Health and Practice Project
EPOC: Effective Practice and Organisation of Care
ITT: Intention to treat
NRS: Non-randomised study
PICO: Population, Intervention, Comparison, Outcome (review scope)
RCT: Randomised controlled trial
RoB: Risk of bias
ROBINS-I: Risk of Bias In Non-Randomised Studies of Interventions
RP: Review protocol
SQ: Signalling question
SS: Study specific
